# The Influence of Electrode Thickness on the Structure and Water Splitting Performance of Iridium Oxide Nanostructured Films

**DOI:** 10.3390/nano12193272

**Published:** 2022-09-21

**Authors:** Abeer S. Altowyan, Mohamed Shaban, Khaled Abdelkarem, Adel M. El Sayed

**Affiliations:** 1 Department of Physics, College of Science, Princess Nourah bint Abdulrahman University, P.O. Box 84428, Riyadh 11671, Saudi Arabia; 2 Physics Department, Faculty of Science, Islamic University of Madinah, P.O. Box 170, Madinah 42351, Saudi Arabia; 3 Nanophotonics and Applications (NPA) Lab, Department of Physics, Faculty of Science, Beni-Suef University, Beni-Suef 62514, Egypt; 4 Physics Department, Faculty of Science, Fayoum University, El Fayoum 63514, Egypt

**Keywords:** IrOx films, nanorod morphology, water splitting, solar-to-hydrogen conversion

## Abstract

For a safe environment, humanity should be oriented towards renewable energy technology. Water splitting (WS), utilizing a photoelectrode with suitable thickness, morphology, and conductivity, is essential for efficient hydrogen production. In this report, iridium oxide (IrO_x_) films of high conductivity were spin-cast on glass substrates. FE-SEM showed that the films are of nanorod morphology and different thicknesses. UV-Vis spectra indicated that the absorption and reflectance of the films depend on their thickness. The optical band gap (*E*_g_) was increased from 2.925 eV to 3.07 eV by varying the spin speed (SS) of the substrates in a range of 1.5 × 10^3^–4.5 × 10^3^ rpm. It was clear from the micro-Raman spectra that the films were amorphous. The *E*_g_ vibrational mode of Ir–O stretching was red-shifted from 563 cm^−1^ (for the rutile IrO_2_ single crystal) to 553 cm^−1^. The IrO_x_ films were used to develop photoelectrochemical (PEC) hydrogen production catalysts in 0.5M of sodium sulfite heptahydrate Na_2_SO_3_**·**7H_2_O (2-electrode system), which exhibits higher hydrogen evaluation (HE) reaction activity, which is proportional to the thickness and absorbance of the used IrO_x_ photocathode, as it showed an incident photon-to-current efficiency (*IPCE*%) of 7.069% at 390 nm and −1 V. Photocurrent density (*Jph* = 2.38 mA/cm^2^ at −1 V vs. platinum) and PEC hydrogen generation rate (83.68 mmol/ h cm^2^ at 1 V) are the best characteristics of the best electrode (the thickest and most absorbent IrO_x_ photocathode). At −1 V and 500 nm, the absorbed photon-to-current conversion efficiency (*APCE*%) was 7.84%. Electrode stability, thermodynamic factors, solar-to-hydrogen conversion efficiency (*STH*), and electrochemical impedance spectroscopies (EISs) were also studied.

## 1. Introduction

Photoelectrochemical water splitting (WS) for hydrogen production plays a crucial role in renewable (sustainable and eco-friendly) energy technology, where humanity should be oriented to decrease reliance on fossil fuels that harm the environment [1,2,3]. In the WS process, the film’s thickness, morphology, and conductivity can affect the performance of the photoelectrode (catalyst) [4]. The thickness affects the light permeability across the film. Increasing the light absorption efficiency is decisive in the overall solar to *H*_2_ conversion efficiency where the photo-generated carriers perform the hydrogen evaluation (HE) reaction and the oxygen evolution (OE) reaction, considering that the HE reaction is a simple process (two electron-transfer reactions) compared with the OE reaction that involves four electron–proton-coupled reactions and consumes a higher amount of energy [5,6,7]. Although the ultralow-earth availability, no low-priced and convenient replacement for the Ir-based catalyst has been proposed so far [8]. IrO_x_ is one of the few materials that can remain stable under harsh acidic media where most other metal oxides corrode. IrO_x_ proved to be the most promising photocatalyst for high-efficiency solar WS devices, where it has shown the smallest over-potential (˂0.5 V) [1]. Additionally, the unique advantages of IrO_2_ include excellent sensitivity, electronic structure, atom arrangement, bonding geometry, and appropriate electric conductivity. The wonderful biocompatibility makes it attractive as a bio-stimulating electrode for implantable bio-medical and pH-sensing devices [9,10,11].

Two-dimensional materials (thin films) are highly favorable for surface-sensitive reactions due to their nearly complete utilization, i.e., the surface-active sites are of maximized density [12]. Ir oxides are usually divided into two types: anhydrous crystalline and highly defective amorphous (hydrous). The amorphous (IrO_x_) with a high surface concentration of Ir^3+^, electrophilic oxygen ($O^{-}$), and active sites, combined with the high level of bulk defects, shows a higher OE reaction activity (at least an order of magnitude larger) relative to crystalline IrO_2_, in which only 1–2% of the Ir atoms participate in the reaction [6,8]. Various physical and chemical approaches have been applied to prepare IrO_2_ or IrO_x_ thin-film electrodes with exceptional activity and stability [3]. The physical methods include the sputtering technique, thermal oxidation, pulsed laser deposition (PLD), and atomic layer deposition (ALD). These techniques are based on converting the metallic Ir to IrO_2_, which is a difficult (complex and costly) process, besides the volatility of nonstoichiometric iridium oxides [9]. By controlling the substrate temperature and using O_2_ or H_2_O as atmospheric gases, Ito et al. [13] prepared amorphous and crystalline iridium oxides using the sputtering technique, for electrochromic device applications. Yet, besides the poor adhesion and the possible damage to the substrate due to the ion collision, the sputter system’s design affects the film’s quality, which requires a complicated high-vacuum system. In addition, it is not suitable for electrodes with a large surface area due to undesirable crack formation [7,11]. Pan et al. [10] utilized the potent oxidative characteristics of the molten Li_2_CO_3_ to thermally oxidize the Ir film in a reactor at a high temperature (750 °C), resulting in an IrO_x_ electrode with a dense and homogeneous surface made up of cone-shaped nanoparticles. Hou et al. [9] fabricated IrO_2_ thin films on TiO_2_ substrates by PLD at 500 °C and 100 mTorr O_2_ pressure. Matienzo et al. [14] prepared IrO_2_ and NiO thin films (˂60 nm in thickness) on Ni substrates using the ALD technique. These crystalline films showed good activity for the OE reaction, but only under high temperatures and high pHs.

The reported chemical methods include sol–gel deposition, electrochemical, electrodeposition, and chemical bath deposition (CBD), which are cost effective, low temperature, and yield films of controlled thickness, density, crystallinity, and oxygen content. Chung et al. [11] used CBD to prepare IrO films of excellent charge storage capacity, charge injection capability, and magnificent biocompatibility useful for biomedical device applications. Korkmaz et al. [15] fabricated GO/IrO_2_ films on glass, PMMA, FTO, and ITO substrates by CBD to act as supercapacitors for energy storage applications. These compositions achieved maximum capacitance of 551.7, 837.7, 433.2, and 569.7 F/g, respectively. XRD showed that the first two films were amorphous and the other two were of polycrystalline structure. Sachse et al. [16] prepared mesoporous IrO_x_ films of 64–79 nm thickness on Si and glass substrates using the sol–gel dip-coating method, using (Ir(CH_3_COO)_n_, 48% Ir) dissolved with a copolymer (polyethylene oxide–polybutadiene) in ethanol.

One of the most interesting and facile chemical methods is the sol–gel spin casting method. Guan et al. [17] expatiated this technique to prepare amorphous IrO_x_ films by casting the H_2_IrCl_6_/(polyvinyl pyrrolidone) PVP on FTO substrates followed by annealing at 300 °C and air plasma treatment. They reported excellent catalytic performance towards OE reaction in 0.5 M H_2_SO_4_ electrolyte at room temperature (RT) with an overpotential of 0.291 V@10 mA/cm^2^, a Tafel slope of 0.0554 V/dec, and ultrahigh mass activity of 993 A/g at 1.55 V. Chandra et al. [5] prepared IrO_x_ and IrO_2_ thin films by spin casting of the K_2_IrCl_6_ solution onto FTO-coated glass substrates. The film annealed at 300 °C was IrO_x_ (OH)_y_ and showed a low overpotential of 0.24 V and a Tafel slope of 0.042 V/dec at pH = 7, which is comparable with the value for IrO_x_·nH_2_O film (0.04–0.05 V/dec). Moreover, the spin-casting process allows us to control the films’ thickness by adjusting the solution molarity, repeating the coating/pre-heating step or spin-casted layers, and choosing the number of rotations per minute (rpm) or the spin speed (SS). Previously, it was reported that the film thickness (*d*) is inversely proportional to $\sqrt{rmp}$, i.e., $\sqrt{SS}$ [18]. The film’s thickness can be evaluated accurately by X-ray fluorescence (XRF) spectroscopy and cross-sectional scanning electron microscopy (SEM) [4].

It has been a problem for a while to find the optimal electrode thickness. IrO2 with a thickness of 23–84 nm was used as the bottom electrode by Shimizu et al. [19] to investigate the electrical characteristics of PZT thin films. They discovered that the thick electrode performed well as a good barrier for the elements Pb, Zr, Ti, and O and that an intermediate amorphous layer was formed to serve as a diffusion barrier layer between PZT and the electrode. Additionally, Sardarinejad et al. [20] reported that among the R.F. sputtered RuO2 films used as pH sensors (thickness in a range of 50–425 nm), the film with a thickness of 300 nm displayed the best sensitivity of 68.63 mV/pH, steady output potentials for all pH values in the range of 2–12, fast response, good stability, and reversibility. Liu et al. [21] recently created 2D Ni_3_(hexaiminotriphenylene)_2_ films with one to four layers and discovered that the film with three layers had the best OER performance and maximum stability during 103 CV cycles. IrOx is widely thought to be one of the best OER catalysts. However, because IrOx has a low cathodic current compared to platinum, it is not very often looked into for its HER activity. Herein, IrOx exhibits higher HE reaction activity and, as mentioned above, the amount of electrode material or thickness can influence the catalytic activity and stability of IrO_x_ films, i.e., its solar to *H*_2_ conversion efficiency, which is less investigated. This work aims to prepare IrO_x_ films by sol–gel spin casting with different thicknesses, to account for the influence of SS and *d* values on the structure of IrO_x_ and the related optical properties and photocatalytic activity through the WS process.

## 2. Experimental Section

### 2.1. Materials and Preparation

Iridium (III) chloride hydrate (IrCl_3_.xH_2_O, 54.1% Ir, molecular weight = 298.58 g/mol., Aspira Chemica) was used as the Ir source. Absolute ethanol and acetic acid (CH_3_COOH) were served as solvents and chelating agents, respectively. The solution molarity was fixed at 0.035 and the determined amount of IrCl_3_.xH_2_O was dissolved in 10 mL of ethanol by utilizing a magnetic stirrer for 2 h @50 °C. The solution was stirred for a few minutes before acetic acid was added. Prior to spin casting, the solutions were matured for more than 20 h. Cleanup was done on the glass substrates with detergent acetone and ethanol in an ultrasonic bath, in separate steps for 10 min for each and finally air dried. The IrO_x_ solution was spin cast at the SS of the substrate in a range of SS = 1.5 × 10^3^–4.5 × 10^3^ rpm for 30 s. After each coating, the substrates were pre-annealed at 200 °C for 10 min to remove any leftover solvent and volatile compounds. The casting and drying procedure was completed six times. The obtained samples were given the names SS1.5, SS2.5, SS3.5, and SS4.5, which corresponded to SS = 1.5 × 10^3^, 2.5 × 10^3^, 3.5 × 10^3^, and 4.5 × 10^3^ rpm. The final annealing procedure took done for an hour at 500 °C in a ceramic air furnace.

### 2.2. Characterization and Photocatalytic Performance

The films’ surface morphology and thickness evaluation were analyzed using FE-SEM (model: ZEISS SUPRA 55 VP and ZEISS LEO, Gemini Column). Raman data were recorded using the spectrometer of model: i-Raman Plus from B&W Tek of high sensitivity portable, in a range of 200–1450 cm^−1^. The films’ chemical composition was investigated by Energy Dispersive X-ray Spectrometer (JED-2300T, JEOL). The optical absorption spectra were recorded at RT by the double-beam Shimadzu spectrophotometer (UV/VIS/NIR 3700) in a wavelength range of 200–1800 nm. The photocatalytic activity measurements were performed by OrigaFlex potentiostat (OGFEIS linked to an OGF500 Pack, Rillieux-la-Pape, France) in 100 mL of 0.5 M (Na_2_SO_3_**·**7H_2_O) solution at RT with the nanocomposite electrode with a 1 cm^2^ surface area as the photocathode (working electrode), and a Pt-electrode of the same area as the counter electrode (auxiliary electrode). The simulated solar light was incident on the electrode surface with a standard white illuminance (AM 1.5 G, 100 mW/cm^2^) provided by a mercury xenon light source (Newport, MODEL: 66926-500HX-R07, Newport, Oxfordshire, UK).

## 3. Results and Discussion

### 3.1. Film Morphology and Thickness Evaluation

**Figure 1:**
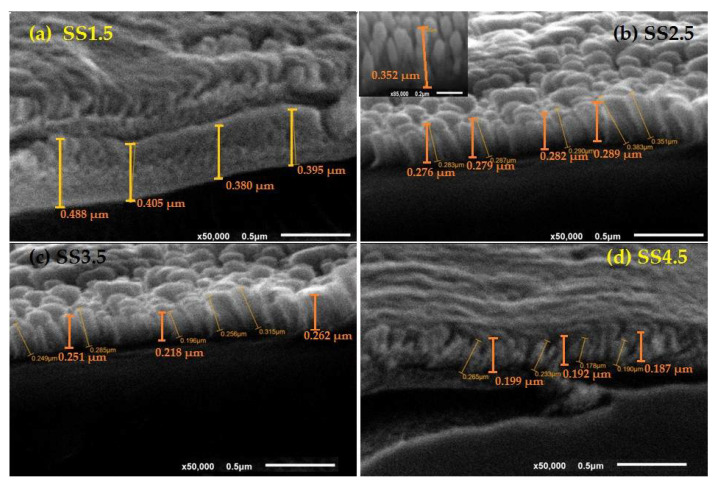
(**a–d**): FE-SEM image of IrOx films spin deposited at spin speed (SS) of (**a**) (1.5–4.5) × 10^3^ rpm. The inset of (**b**) shows the nanorod morphology (scale bar 0.2 µm and magnification ×50,000).

FE-SEM microscope was used to study the surface morphologies and the dependence of the thickness of the film on the SS or the number of rpm for the substrate during the deposition process. Figure 1 shows the cross-sectional SEM images of films spin casted at SS in a range of 1.5 × 10^3^–4.5 × 10^3^ rpm. The surface of the films is well covered with IrO_x_ of nanorod morphology. The density of the nanorods reduced and the uniformity improved with increasing SS value. Moreover, increasing the SS from 1.5 × 10^3^ rpm to 4.5 × 10^3^ rpm results in a continuous reduction in the average thickness (*d_av_*_._) of the films from 0.417 µm to 0.193 µm according to Table 1. The values of $d_{av.}/{\left(SS\right)}^{-1/2}$ are in a range of 13.78–16.04, which can be considered constant. Therefore, one can conclude that the deposited amount of IrO_x_ is inversely proportional to $\sqrt{SS}$, which is consistent with the previously published data [18].

### 3.2. Optical Properties and Raman Spectra

**Figure 2:**
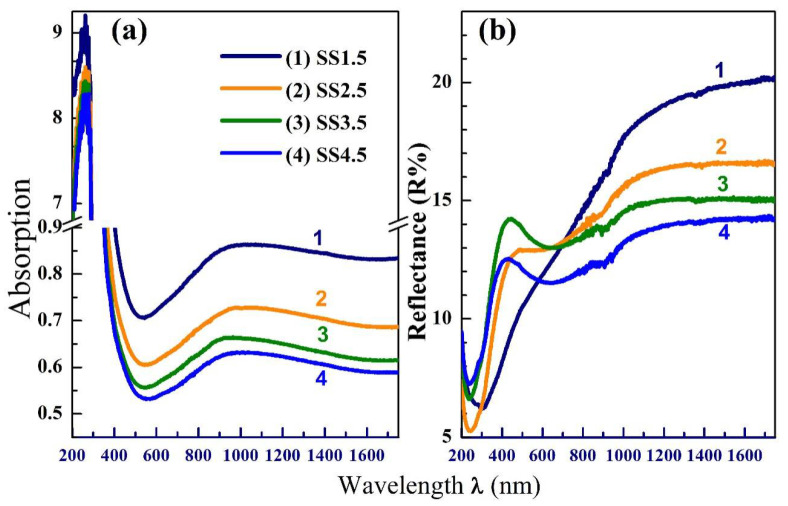
The optical absorbance (**a**) and reflectance spectra (**b**) of the spin-casted films at different SS.

The films exhibit strong absorption (Abs.) at very low wavelengths associated with an absorbance band at *λ* ≈ 270 nm, Figure 2a. This band is commonly found in the absorption spectra of the nano-sized metal (M) oxides and owing to the M–O band [22]. The Abs. sharply decreases with increasing *λ* till about 544 nm, then increases till 925 nm. After that, it reaches nearly a constant value at higher wavelengths. As seen, increasing the metal loading via improving the IrO_x_ thickness, by decreasing SS from 4.5 × 10^3^ rpm to 1.5 × 10^3^ rpm, yields enhanced Abs. Figure 2b indicates that the reflection changes in an unordered manner with thickness but is proportional to the film thickness at *λ* ˃ 700 nm. This indicates the possible use of these films in the sensing application for IR radiation.

**Figure 3:**
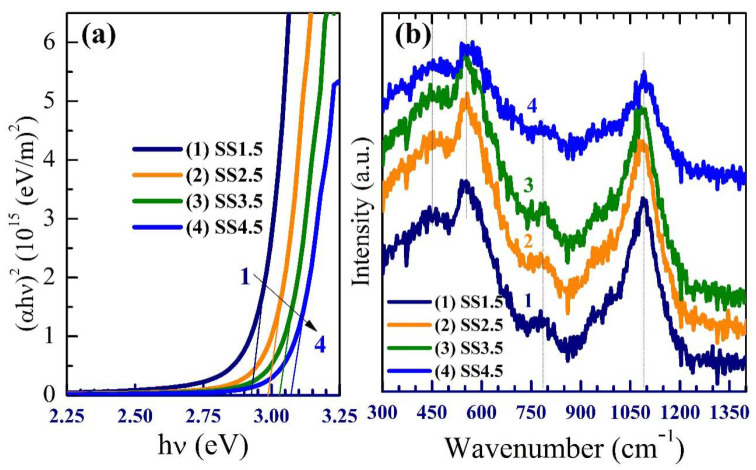
Tauc’s plots for *E*_g_ determination (**a**), and the Micr-Raman spectra (**b**) of IrOx films spin casted at different SS.

To account for the optical band gap (*E*_g_) of the films, the absorption coefficient *α* was determined (*α* = Abs./film thickness) and then introduced in the Tauc’s relation (*αhυ*)^2^ = *C*(*hυ* − *E*_g_), where the incident energy of electromagnetic photons; *hυ =* 1242/*λ*, and C is a constant. Extrapolating the linear parts of (*αhυ*)^2^ and *hυ* curves to the x-axis, Figure 3a, provides the *E*_g_ values. As seen, the *E*_g_ of the films is affected by their thickness, where it increases from 2.925 eV to 3.07 eV with decreasing the thickness or increasing *SS* from 1.5 × 10^3^ to 4.5 × 10^3^ rpm. Similarly, increasing the solution flow rate and deposition time during the spray deposition process for CdS thin films yields thick films with lower *E*_g_ values [23]. The obtained *E*_g_ values (2.925–3.07 eV) represent the separation between the t_2g_ and e_g_ sub-levels of the Ir 5d band [16]. The *E*_g_ of the IrO_2_ single crystal is 3.5 eV. These lower *E*_g_ values are a result of the amorphous nature of our IrOx films and the poorly defined band edges, which cause disorder-induced tails to extend into the band gap.

Raman spectroscopy is a powerful technique to investigate the lattice vibrations and the structural evidence about the materials. In the literature, the *E*_g_ vibrational mode of IrO_2_ with the rutile structure, originating from Ir–O stretching, appears at 563 cm^−1^. Figure 3b shows the Raman spectra of SS1.5–SS4.5 films. As seen, the *E*_g_ mode appears at ~553 cm^−1^, indicating deviation in our IrO_x_ films regarding that of an IrO_2_ single crystal. This redshift is related to the mixed-valence states for iridium in IrO_x_ [24]. Further, this shift can be a result of the stress effect between IrO_x_ and the substrate and it has been observed that it is minimized as the coating becomes more crystalline [25]. The broadness of this band (extending from 510–670 cm^−1^) indicates a lack of crystallinity (amorphous films) or poor crystallinity [26] The weak and broad peak at 762 cm^−1^ is ascribed to the *A*_1*g*_ mode. Gao et al. [2] detected Ir–O vibrations at *E*_g_, A_1g_ and B_2g_ modes for IrO_2_ and amorphous Li-doped IrO_x_ at 540 and 710 cm^−1^, respectively. Saeed et al. [27] reported that the peak position of Ir–O–Ir stretching is due to the Ir^3+^ species appearing at 608 cm^−1^. Pavlovic et al. [28] predicted a vibrational mode for the Ir = O stretching between 771 and 829 cm^−1^. Finally, the sharp peak at 1092 cm^−1^ originated from the 2ed-order of $2A_{1}^{LO}$ mode [29]. The sharpness of this band reduced with reducing the film thickness or increasing SS from 1.5 × 10^3^ to 4.5 × 10^3^ rpm. These results verify the amorphous nature of the prepared films and the influence of film thickness on their microstructure.

## 4. Photoelectrochemical (PEC) Water Splitting Measurements

### 4.1. Photoelectrochemical Behaviour of IrO_x_, Stability, Number of Hydrogen Moles, and the Benchmark Efficiency

**Figure 4:**
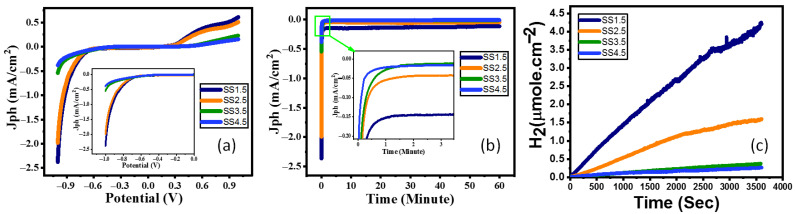
(**a**) Variation in current density vs. applied voltage for all photocathodes in standard white-light luminance, (**b**) variation in current density vs. exposure time for all photocathode @−1 V current density, and (**c**) number of hydrogen moles versus production time for all photocathodes in white-light luminance.

The products of water splitting (such as hydrogen and oxygen) can be precisely measured using gas chromatography (GC); however, in this case, we performed potentiometry and amperometry measurements under white light and monochromatic light illumination to assess the photoelectrode’s catalytic activity [30]. The photoelectrochemical properties of the IrO_x_ photocathodes were measured under a standard white illuminance (AM 1.5 G, 100 mW/cm^2^) and evaluated with the use of a 400 W mercury xenon light source (Newport, MODEL: 66926-500HX-R07, Newport, Oxfordshire, UK). The OrigaFlex potentiostat (OGFEIS linked to an OGF500 Pack, Rillieux-la-Pape, France) was used to obtain all measurements. We used 0.5 M (Na_2_SO_3_**·**7H_2_O) as an electrolyte to avoid sample degradation in both acidic and alkaline electrolytes, with IrO_x_ films as the working electrode, while a platinum electrode was used as the auxiliary electrode. The working electrode and the auxiliary electrode were dipped in the 0.5 M (Na_2_SO_3_**·**7H_2_O) electrolyte. The Jph–V characteristics illustrate that the largest Jph values can be found in the negative voltage range, which means that the electrodes are made of P-type (photocathode) semiconductors with the majority of free carriers being holes. The photoelectrochemical current density (Jph) is shown in Figure 4a to be affected by the applied voltage as it changes from −1 V to +1 V. The photoelectrochemical Jph rose for all photoelectrodes when the negative applied voltage was raised. The photocurrent density enhanced as the SS was reduced and the thickness increased, as shown in Figure 4a. This might be owing to the expansion of the optical bandgap into the visible light range as a result of the increasing SS from 2.925 eV to 3.07 eV. At −1 V, SS1.5 photocathode produced a maximum Jph of 2.38 mA/cm^2^ when compared to SS2.5, SS3.5, and SS4.5 photocathodes (1.98, 0.54, and 0.38 mA/cm^2^ at −1 V, respectively). This indicates that the current density improved by decreasing SS and the bandgap of IrO_x_ photocathodes while increasing absorption. The change in current density vs. time is seen in Figure 4b. Within 60 s, the current density had plummeted to around 0.15, 0.05, 0.022, and 0.019 mA/cm^2^ for each of the SS1.5, SS2.5, SS3.5, and SS4.5 photocathodes, respectively. Despite the early reduction in photocurrent density, there is a minor drop in current density at periods longer than 60 s until it approaches constant values of roughly 0.124, 0.44, 0.015, and 0.01 mA/cm^2^ for each of the SS1.5, SS2.5, SS3.5, and SS4.5 photocathodes, respectively. Further, the stability of the SS1.5 (Thick, 417 nm) and SS4.5 (Thin, 193 nm) electrodes is tested as a function of the number of runs (10 runs). The data are provided in Figure S1 (Supplementary Data), which shows that the SS1.5 retains ~95.0% of its efficiency versus 65.8% for SS4.5 after 10 runs. This clearly showed that SS1.5 is more stable than the SS4.5 electrode. This demonstrates that the optimized IrOx film is extremely stable and may be used as photocathodes in the hydrogen-splitting process for a long period.

Faraday’s equation, Equation (1), was used to calculate the number of moles of hydrogen generated by the photoelectrochemical WS technique [31].

(1) $$H_{2}\left(moles\right)=\int_{0}^{t}\frac{Jph}{F}\;dt$$

where *Jph* is photocurrent density, *F* is the Faraday constant (96,500 C/mol), and *t* is the period. The ratio of *H*_2_ moles generated as a function of generation time is plotted in Figure 4c using the reported *Jph*-time data in Figure 4b. The estimated hydrogen output rate was 83.68, 32.93, 7.06, and 5.47 mmole/h.cm^2^ for each of the SS1.5, SS2.5, SS3.5, and SS4.5 photocathodes, respectively. The solar-to-hydrogen conversion efficiency (*STH*) is the ratio of total hydrogen energy output to total sunlight energy input. Equation (2) [32] is applied to find the total efficiency of the PEC water splitting cell:

(2) $$STH=\left[\;\left(H_{2}/S\right)\times \;\mathrm{QUOTE}\;\left(237\;KJ/mol\right)]/[P_{total}\;\mathrm{QUOTE}\;\times A\right]$$

where *H_2_/S* is the rate of *H*_2_ generation per second, *P_total_* represents the total power density of the incident light (mW/cm^2^), and *A* is the photoelectrode area. The estimated *STH* was 2.97, 1.17, 0.25, and 0.19% for each of the SS1.5, SS2.5, SS3.5, and SS4.5 photocathodes, respectively. According to the results, the SS1.5 electrode with the lowest bandgap and highest absorption is the best photocathode for hydrogen evolution.

### 4.2. PEC Behaviour of the SS1.5 Photocathode under the Effect of Monochromatic Light Illumination and Photoelectrochemical Efficiencies

**Figure 5:**
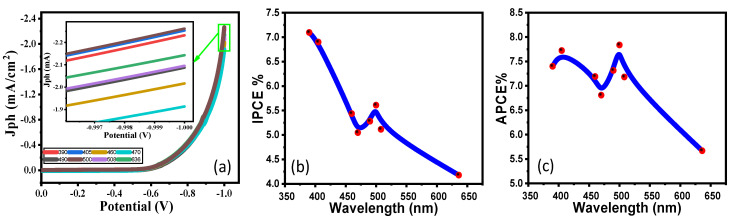
SS1.5 photocathode: (**a**) variation in current density vs. the applied voltage under monochromatic luminance, (**b**) *IPCE*% and (**c**) ABCE% versus the incident wavelength at −1 V.

In 0.5 M (Na_2_SO_3_**·**7H_2_O) at RT, bandpass filters of wavelength ranging from 390 to 636 nm have been utilized to investigate the SS1.5 photocathode’s response to monochromatic light and to evaluate its efficiencies in water splitting process for hydrogen generation. According to Figure 5a, the maximum photocurrent was measured at 500 nm and −1 V and was determined to be *Jph* = 2.26 mA.cm^−2^, while the lowest photocurrent was determined to be *Jph* = 1.91 mA.cm^−2^ at 470 nm. From 390 to 636 nm, the SS1.5 photocathode’s current values are shown to be in a small range. This current density–wavelength range dependency may be related to the SS1.5 photocathode’s absorption behavior at each wavelength, as at 544 nm, SS1.5 has the lowest absorbance and has good absorbance as wavelength changes after and before that value, which confirms the PEC catalytic response of the optimum photoelectrode for the *H*_2_ production process. Generally, this shows that the SS1.5 photocathode is sensitive to a lot of the sun’s light and is good at absorbing a lot of it in the visible range. The SS1.5 photoelectrode’s improved solar absorption and application to efficient *H*_2_ generation from H_2_O splitting are further demonstrated by estimating the external quantum efficiency or incident photon-to-current conversion efficiency (*IPCE*%). At various wavelengths in Figure 5a, Equation (3) [31] is used to estimate the *IPCE*% at a fixed voltage of −1 V:

(3) $$IPCE\%=1240\times \frac{Jph}{\left(\lambda .P\right)}\times 100\%$$

where *λ* is the wavelength of the incident photons and *P* is the illuminating light power density of the Xenon lamp as a function of the monochromatic light wavelength. The change in *IPCE*% with wavelength is represented in Figure 5b. At 390 nm, the maximum *IPCE*% of the SS1.5 photoelectrode is obtained. At 390 nm, it was 7.96% and at 500 nm, it was 5.61%, with an *IPCE*% of 4.18% at 636 nm being the lowest. The influence of optical losses, such as transmission (*Tr*) or reflection (*R*), was still not considered in the *IPCE*% computations. To compensate for optical losses, the internal quantum efficiency, also known as the absorbed photon-to-current conversion efficiency (*APCE*%), is calculated. The photocurrent generated by each absorbed photon is made up of the number of PEC-generated carriers. *APCE*% is calculated using Equation (4) [33,34]:

(4) $$APCE\left(\lambda \right)=\frac{IPCE\left(\lambda \right)}{A\left(\lambda \right)}=\frac{IPCE\left(\lambda \right)}{1-R-T_{r}}\;\;\;$$

where *A*(*λ*) denotes optical absorption.

The change in *APCE%* versus incident wavelength is illustrated in Figure 5c. This graph shows two significant *APCE%* values: 7.84% around 500 nm and 7.72% around 405 nm, with the lowest value being 5.67% at 636 nm. These findings support the observation in Figure 2 that absorbance falls significantly as wavelength increases until around 544 nm, then increases until 925 nm. As we noted in the calculation of the applied bias photon-to-current efficiency (ABPE) for the employed electrodes, Figure S2 (Supplementary Data), the optimal applied potential for the best PEC performance should alter with the change in electrode thickness [35,36]. As the film thickness increases, the optimum potential value decreases [37]. Based on the highest recorded value of ABPE, the optimum potential is reduced from 0.86 V (SS4.5) to 0.71 V (SS1.5) by rising the film thickness from 193 to 417 nm.

### 4.3. Effect of Temperature and Thermodynamic Parameters

**Figure 6:**
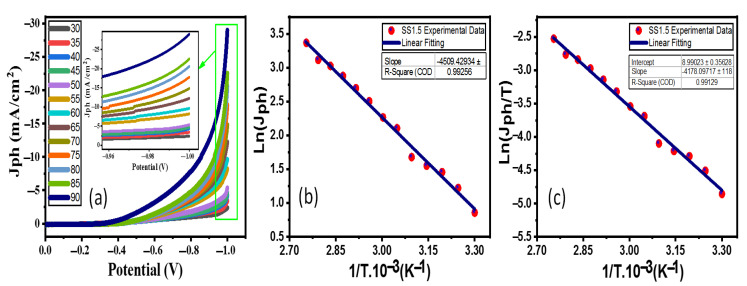
Effect of temperature for the SS1.5; (**a**) the change in *J_ph_*-V behaviors at distinctive temperatures, (**b**) *Ln* (*J_ph_*) versus (*T*^−1^), and (**c**) *Ln* (*J_ph_*.*T*^−1^) versus (*T*^−1^).

If you increase the temperature in the SS1.5 photoelectrodes in the 0.5 M (Na_2_SO_3_·7H_2_O) electrolyte from 30 °C to 90 °C, the PEC Jph-voltage of the photoelectrodes changes. This is shown in Figure 6a. The Jph significantly improved from 2.36 mA/cm^−2^ to 28.99 mA/cm^−2^ when the temperature was raised to 90 °C at −1 V. This significant rise in Jph with the rise in *T* can be explained this way: (i) There would be more electrons and holes in the conduction and valence bands, respectively, if the photogenerated carriers had higher *Ts*. This would speed up redox reactions and *Jph*, which is how quickly electrons and holes move around. (ii) In the equation *μ*= *q*$\tau_{n}$/m*, where *μ* is the charge carriers’ mobility, *q* total charge of charge carriers,$\;\tau_{n}$ is the carrier’s lifetime, and m* is its effective mass, increasing *T* would make it easier for charge carriers to move around as it increases charge carriers’ mobility. This would make the charge carriers last longer as their lifetime is increased. (iii) The minority carrier diffusion length is enhanced by increasing the *Jph*, which is directly proportional to the square root of the absolute *T* based on the relationship: $Jph\;\alpha \;L_{diff}=\sqrt{\mu \;\frac{k_{B}T\;}{q}\;\tau_{n\;}}$ [38]. Additionally, thermodynamic factors, such as activation energy (*E_a_*), enthalpy (ΔH*), and entropy (ΔS*), must be evaluated. Figure 5b illustrates the connection between the reciprocal of the absolute *T* (1/*T*) and *Jph* (rate of reaction) for the SS1.5 electrodes. The Arrhenius Equation (5) [39] is used to determine the value of *E_a_* based on the linear fitting slope seen in Figure 5b.

(5) $$Ln\;\left(J_{ph}\right)=-\frac{E_{a}}{R}\left[\;\frac{1}{T}\;\right]$$

where *R* = 8.314 J/(K.mol), the universal gas constant. According to Figure 6b, slope equals −*E_a_/R* and the SS1.5 photoelectrode’s *E_a_* value is 37.473 kJ/mol. The values of ΔH* and ΔS* for the *H*_2_ production process are also computed using the Eyring equation by charting the relationship *Ln* (*Jph*/*T*) against (1/*T*) in Figure 6c. The Eyring equation is denoted by (6) [33]:

(6) $$Ln\frac{J_{ph}}{T}=-\frac{\Delta H^{*}}{R}.\;\frac{1}{T}+Ln\left(\frac{K_{B}}{h}\right)+\frac{\Delta S^{*}}{R}$$

where *K_B_* = 1.38 × 10^−23^ J/K, the Boltzmann’s constant, and *h* = 6.626 × 10^−34^ J.s, the Planck’s constant. The linear fitting’s slope indicates that the ΔH* value for SS1.5 is 34.736 kJ/mol and the intercept indicates that the ΔS* value is −122.79 J.mol^−1^.K^−1^.

According to Table 1, the PEC performance in this work was evaluated against several photoelectrodes that had previously been published. The reported *J_ph_*, *IPCE%*, and *APCE%* values confirmed that SS1.5 is efficient PEC electrode for WS in visible light. Thus, it has been determined that the SS1.5 electrode is very suitable for PEC hydrogen generation.

### 4.4. PEC Impedance Spectroscopy (PEC-IS)

**Figure 7:**
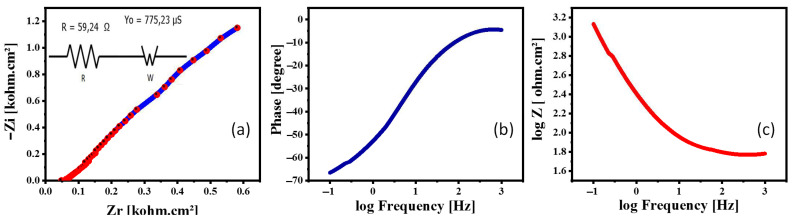
The SS1.5 photocathode impedance in 0.5 M (Na_2_SO_3_**·**7H_2_O) electrolyte at RT under white-light illumination; (**a**) the Nyquist Z plot and the equivalent circuit, (**b**,**c**) Bode plots: (**b**) the variation in phase with frequency and (**c**) the change in total impedance with frequency.

In all electrochemical processes, electrochemical impedance spectroscopy (EIS) has become a highly essential research technique. The electrochemical interphase is frequently characterized by an equivalent circuit applicable to the conditions of the experiment, utilizing circuit parts that reflect the numerous physical processes involved, as in the interpretation of impedance data. The charges transport between the active photoelectrode and the electrolyte contact determines the photoelectrochemical system’s impedance. The Warburg impedance, which simulates semi-infinite linear diffusion—that is, diffusion in one dimension that is only constrained on one side by a planar electrode—is the simplest and most frequent circuit element for modelling diffusion behavior. The Warburg impedance generates a nearly straight line with a phase of 45° in the Nyquist plot, which is extremely noticeable in EIS. When you see a 45° line on the Nyquist plot, it typically means diffusion. To explore the charge carriers’ dynamics of the optimized SS1.5 photocathode, PEC-IS data were obtained at RT using an OrigaFlex potentiostat (OGFEIS linked to an OGF500 Pack, Rillieux-la-Pape, France). Under white-light illumination, PEC-IS data were made in a frequency (f) ranging from 100 mHz to 1 kHz. Figure 7a shows a Nyquist plot of SS1.5 submerged in 0.5 M (Na_2_SO_3_·7H_2_O). The results are also shown in the Bode plots (Figure 7b,c). The Warburg equivalent circuit was utilized to simulate the PEC-IS spectra using the OrigaSoft PC Software, as shown in Figure 7a. This analogous circuit of SS1.5 photocathode has a Warburg impedance (W = 775.23 µS) in series with a charge transfer resistance (R_CT_ = 59.24 Ω). These values were the best for the IrO_x_ photocathodes as SS2.5, SS3.5, and SS4.5 photocathodes showed higher charge transfer resistances (110.09, 240.54, and 281.7 Ω) and higher Warburg impedances (645.78, 1851.1, and 2120.6 µS, respectively). This means that R_ct_ and total impedance are decreased with increasing the photoelectrode thickness, as shown in Figures S3 and S4 (Supplementary Data). Similar findings were reported by A.A. Saif and P. Poopalan [40], who found that the impedance of sol–gel Ba_0.6_Sr_0.4_TiO_3_ thin films is inversely related to film thickness. This demonstrates that the optimized electrode can generate a significant quantity of hydrogen. Electron hole recombination, in addition to the charge transfer process (CTP), is the principal controller of the hydrogen evolution reaction (HER). The reordered value of R_CT_ for the SS1.5 electrode is very small, indicating that charges recombination at the electrode/electrolyte interfaces has been significantly decreased, implying that HET has improved [41].

## 5. Conclusions

IrO_x_ thin films with nanorod morphology vertically aligned on glass substrates were successfully spin casted with films thickness in a range of 0.417 µm ≥ *d* ≥ 0.193 µm. UV-Vis-NIR results indicated that changing the SS of the substrates is an effective route to control the films’ absorptivity and reflectivity and the films are a candidate for IR sensing applications. The *E*_g_ values were decreased from 3.07 eV to 2.925 eV by reducing SS from 4.5 × 10^3^ to 1.5 × 10^3^ rpm. Raman spectra illustrated the poor or lack of crystallinity and the mixed-valence state of iridium in IrO_x_. The obtained samples were employed for effective photoelectrochemical hydrogen generation from the water after employing 0.5 M (Na_2_SO_3_**·**7H_2_O) electrolyte and optimizing electrode reusability, applied temperature, and monochromatic-light illumination. Electrode stability, thermodynamic characteristics, conversion efficiencies, amounts of hydrogen moles, and PEC impedance were also assessed and discussed. The SS1.5 photocathode had the greatest photocurrent of 2.38 mA/cm^2^@−1 V, the number of hydrogen moles rate of 83.68 mmol/h.cm^2^, the conversion efficiency of incoming photons to the current (*IPCE%*) of 7.96% @390 nm, absorbed photon-to-current conversion efficiency (*APCE%*) of 7.84% @500 nm, and solar-to-hydrogen efficiency (*STH*) of 2.97% @−1 V. The optimized photoelectrode may be appropriate for industrial applications due to its excellent stability, high conversion efficiency, and inexpensive cost.

## Figures and Tables

**Table 1:** Values of the average thickness (*d_av_*_._) and direct bandgap (*E*_g_) of the IrO_x_ films.

Sample	*d_av_*_._ (µm)	$1/\sqrt{SS}$ $\;(min.^{1/2}Rad^{-1/2})$	$d_{av.}/{\left(SS\right)}^{-1/2}$(µm $\;\cdot min.^{1/2}Rad^{-1/2}$**)**	*E*_g_ (eV)
SS1.5	0.417	0.026	16.04	2.925
SS2.5	0.281	0.020	14.05	3.00
SS3.5	0.244	0.017	14.35	3.03
SS4.5	0.193	0.014	13.78	3.07

## Data Availability

Not applicable.

## Supplementary Materials

The following supporting information can be downloaded at: https://www.mdpi.com/article/10.3390/nano12193272/s1, Figure S1: Reusability test of SS1.5 and SS4.5 photoelectrode.; Figure S2. The variation of applied bias photon-to-current efficiency (ABPE) with the applied potential for the different samples; Figure S3. R_ct_ versus film thickness of the IrO_x_ samples; Figure S4: The total impedance versus frequency for IrO_x_ samples.
