# Supplementary material for: The Influence of Electrode Thickness on the Structure and Water Splitting Performance of Iridium Oxide Nanostructured Films
Source: Nanomaterials (Basel). 2022 Sep 21;12(19):3272. doi: 10.3390/nano12193272 (PMC9565530; doi:10.3390/nano12193272)
Supplement: Supplementary file 1 [file nanomaterials-12-03272-s001.zip › nanomaterials-1894148-supplementary.pdf]

Supplementary data

The Influence of Electrode Thickness on the Structure and Water Splitting Performance of Iridium Oxide Nanostructured Films

Abeer S. Altowyan [1], Mohamed Shaban [2,3,*], Khaled Abdelkarem [3] and Adel M. El Sayed [4,*]

[1] Department of Physics, College of Science, Princess Nourah bint Abdulrahman University, P.O. Box 84428, Riyadh 11671, Saudi Arabia
[2] Physics Department, Faculty of Science, Islamic University of Madinah, P.O. Box 170, Al Madinah Al Monawara 42351, Saudi Arabia
[3] Nanophotonics and Applications (NPA) Lab, Department of Physics, Faculty of Science, Beni-Suef University, Beni-Suef 62514, Egypt
[4] Physics Department, Faculty of Science, Fayoum University, El Fayoum 63514, Egypt
* Correspondence: mssfadel@aucegypt.edu (M.S.); ams06@fayoum.edu.eg (A.M.E.S.)

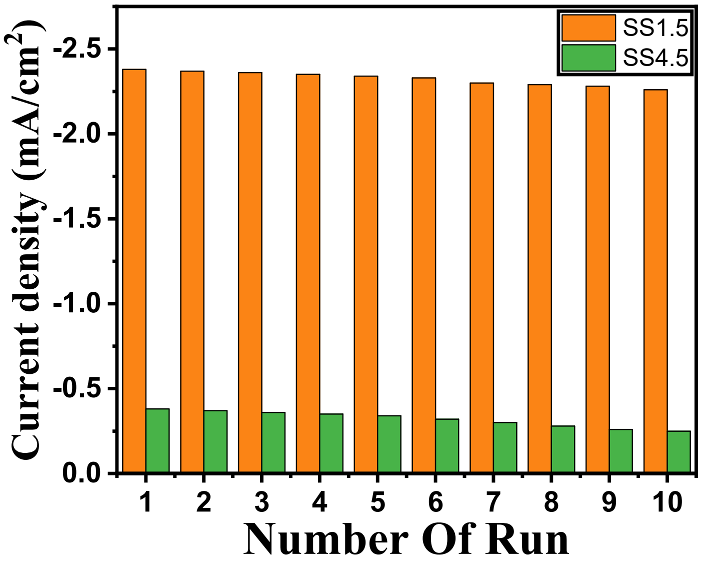

Figure S1. Reusability test of SS1.5 and SS4.5 photoelectrode.

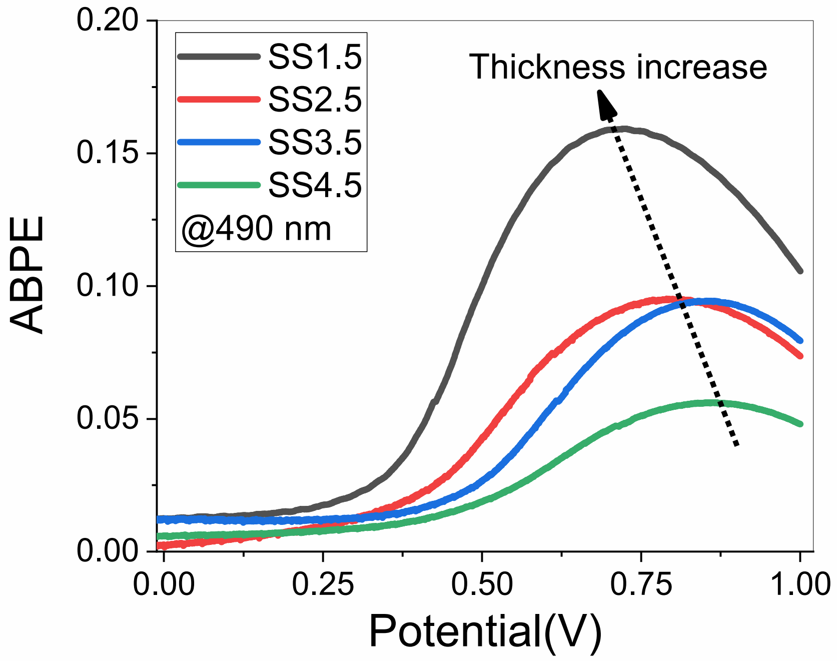

Figure S2. The variation of applied bias photon-to-current efficiency (ABPE) with the applied potential for the different samples. .

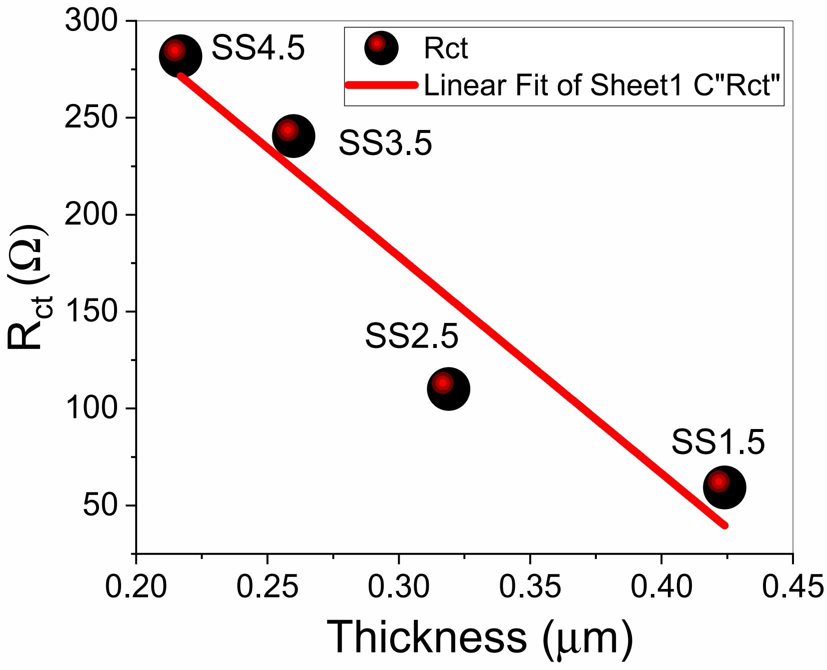

Figure S3. R_{ct} versus film thickness of the IrO_x samples.

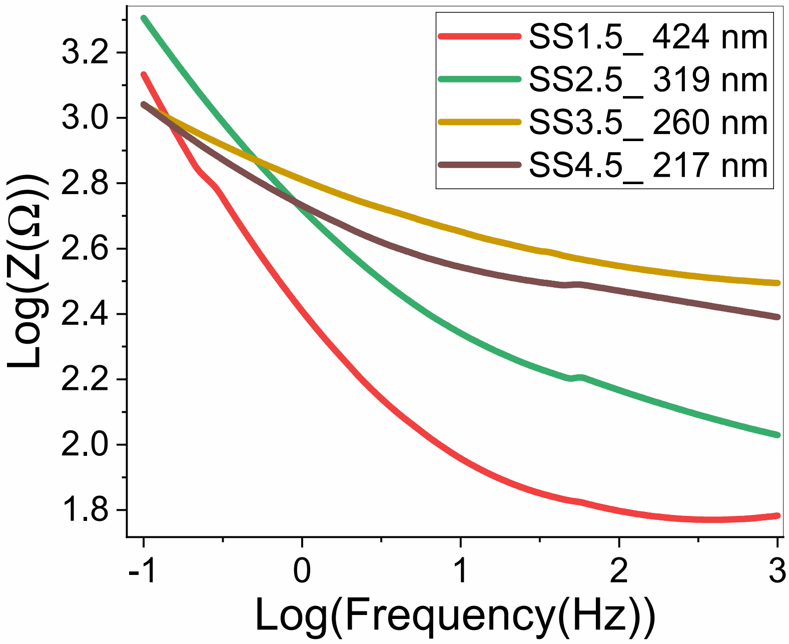

Figure S4. The total impedance versus frequency for IrO_x samples.
